# A systematic review of intravenous gamma globulin for therapy of acute myocarditis

**DOI:** 10.1186/1471-2261-5-12

**Published:** 2005-06-02

**Authors:** Joan L Robinson, Lisa Hartling, Ellen Crumley, Ben Vandermeer, Terry P Klassen

**Affiliations:** 1 Department of Pediatrics, Stollery Children's Hospital and University of Alberta, Edmonton, Alberta, Canada; 2 Alberta Research Centre for Child Health Evidence, Department of Pediatrics, University of Alberta, Edmonton, Alberta, Canada

## Abstract

**Background:**

Intravenous gamma globulin (IVGG) is commonly used in the management of acute myocarditis. The objective of this study was to systematically review the literature evaluating this practice.

**Methods:**

We conducted a comprehensive search (electronic databases, trials registries, conference proceedings, reference lists, contact with authors) to identify studies evaluating the use of IVGG in adults and children with a clinical or histologically proven diagnosis of myocarditis of possible viral etiology and symptoms of less than six months duration. Two reviewers independently screened the searches, applied inclusion criteria, and graded the evidence.

**Results:**

Results were described qualitatively; data were not pooled because only one randomized controlled trial (RCT) with 62 patients was identified. The RCT showed no benefit with respect to cardiac function, functional outcome, or event-free survival. A small, uncontrolled trial (n = 10) showed significant improvement in LVEF from a mean of 24% to 41% 12 months after IVGG in nine survivors. A retrospective cohort study of pediatric patients showed improvement in cardiac function and a trend towards improved survival in patients receiving IVGG (n = 21) versus historic controls (n = 25). Ten case reports and two case series (total n = 21) described improvement in cardiac function after administration of IVGG; two case reports showed no benefit of IVGG. One case of hemolytic anemia was attributed to IVGG.

**Conclusion:**

There is insufficient data from methodologically strong studies to recommend routine use of IVGG for acute myocarditis. Future randomized studies that take into account the etiology of acute myocarditis will be required to determine the efficacy of IVGG.

## Background

Myocarditis is an inflammatory cardiomyopathy that occurs in all age groups [1]. Most cases are thought to be sub-clinical, but myocarditis can manifest as fulminant or chronic heart failure [2]. The inflammation is presumed to most commonly start as an infectious process, although autoimmune and idiopathic forms also occur [1]. Bacterial or protozoal myocarditis is rare in the developed world, with the vast majority of cases being viral [3]. Although most viruses that are pathogenic for humans can cause myocarditis, common etiologic agents are enteroviruses and adenoviruses [4]. It remains unclear if the primary problem in severe disease is ongoing damage from a virus, a post-infectious inflammatory reaction, or a combination of both factors. If ongoing infection is the primary problem, IVGG could be efficacious if it contains antibodies to the microbe. IVGG also has anti-inflammatory properties that prevent the formation of and downregulate cytokines [5] so could be efficacious even if the primary problem is a post-infectious inflammatory reaction.

The objective of this study was to review the evidence evaluating the use of IVGG in the treatment of patients with acute myocarditis. A secondary objective was to determine if there is an identifiable group of acute myocarditis patients (based on age, duration of symptoms, acuity of onset of symptoms, cardiac function at presentation, virologic results, or the presence or absence of histologic evidence of acute myocarditis on cardiac biopsy in patients where a biopsy was performed) who would benefit from IVGG. The review of randomized controlled trials has been registered with the Cochrane Collaboration and regular updates will be available in the Cochrane Library.

## Methods

### Searching

We systematically searched the following electronic databases: Cochrane Central Register of Controlled Trials (4^th ^quarter, 2004), MEDLINE (1966 to January Week 4, 2005), EMBASE (1988 to Week 6, 2005), CINAHL (1982 to January Week 3, 2005), Web of Science (1975 to February 5, 2005), and several trial registries in addition to the Cochrane Heart Group's Trial Registry [6-11]. A comprehensive search strategy was developed by a medical research librarian based on the following terms: immunoglobulin, gammaglobulin, ivig, immunoglobulin, igg, immune, serum, myocarditis, cardiomyopathy, myocardiopathy, carditis, heart, inflammation. The complete search strategy is available from the authors on request. We also contacted the primary author of the RCT, and reviewed the reference lists of all selected articles. In addition, we handsearched proceedings from the following meetings for RCTs: American Heart Association (1999–2004), American College of Cardiology (1998–2005), European Society of Cardiology (1998–2004), and International Heart and Lung Transplantation Society (1998–2004). The search was not limited by language or publication status.

### Study selection

Two reviewers independently screened the searches for potentially relevant studies. The full manuscript of all potentially relevant studies was retrieved and each study was assessed independently by two reviewers for inclusion using predetermined eligibility criteria. Primary studies of any design were eligible for inclusion. Inclusion criteria included reports of patients given IVGG for possible viral myocarditis within six months of onset of symptoms. Because of the poor sensitivity of cardiac biopsy as a diagnostic tool for acute myocarditis, a histologic diagnosis was not required. Exclusion criteria included reports of patients with evidence of ischemic heart disease, rheumatic heart disease, or any other non-viral etiology, onset of myocarditis less than six months postpartum (as the pathogenesis of postpartum myocarditis may differ from that of presumed viral myocarditis), or receipt of immunosuppressive therapy (unless this was not possible as they were reported as part of a case series). Reports of use of high-dose immunoglobulin directed at specific organisms were excluded as efficacy may be influenced by the formulation available and the accuracy of the etiologic diagnosis.

### Assessment of methodological quality

Study quality was assessed independently by two reviewers and discrepancies were resolved by consensus. RCTs were evaluated using the previously validated Jadad 5-point score to assess randomization, double blinding, and losses to follow-up [12]. Allocation concealment was assessed as adequate, inadequate, or unclear [13]. Non-RCTs were graded according to the levels of evidence presented in Table 1.

**Table 1:** Levels of evidence for studies of therapeutic interventions from the Oxford Centre for Evidence-based Medicine (website title ) [14]

Level		Study design
1	A	Systematic review (with homogeneity) of randomized controlled trials
	B	Individual randomized controlled trial (with narrow confidence interval)
	C	All or none
2	A	Systematic review (with homogeneity) of cohort studies
	B	Individual cohort study (including low quality randomized controlled trial, e.g., < 80% follow-up)
	C	"Outcomes" research; ecological studies
3	A	Systematic review (with homogeneity) of case-control studies
	B	Individual case-control study
4		Case series (and poor quality cohort and case-control studies)
5		Expert opinion without explicit critical appraisal, or based on physiology, bench research or "first principles"

### Data extraction

Data were extracted by one reviewer using a standard form and checked for accuracy and completeness by a second. The primary outcome was rate of survival without a transplant or requirement for placement of a left ventricular assist device. Secondary outcomes included change in echocardiographic measures of cardiac function, duration of hospitalization, and improvement in functional symptoms (determined by increased exercise tolerance as measured by any objective test and New York Heart Association Functional Capacity) when available. Data were collected on complications and adverse events.

### Data analysis

Data from studies that were not RCTs were reported but not analyzed further. We planned to combine data from RCTs, to do sub-group analyses, and to test for publication bias, however these were not possible as only one RCT was identified. For the RCT, dichotomous data on efficacy (e.g. event-free survival) were expressed as an odds ratio (OR) with 95% confidence interval. Dichotomous data on adverse events were reported as a risk difference and number needed to harm with 95% confidence intervals. Continuous data (change in left ventricular ejection fraction and peak oxygen consumption) were converted to the mean difference with 95% confidence intervals. For peak oxygen consumption, we assumed a correlation of 0.5 and used the methods of Follmann [15] to calculate the standard deviations of the change from baseline estimates. Since the number per group was not given for this variable, an estimate of the sample size in each group had to be estimated by pro-rating the original group sample sizes to the new total sample size.

## Results

**Figure 1:**
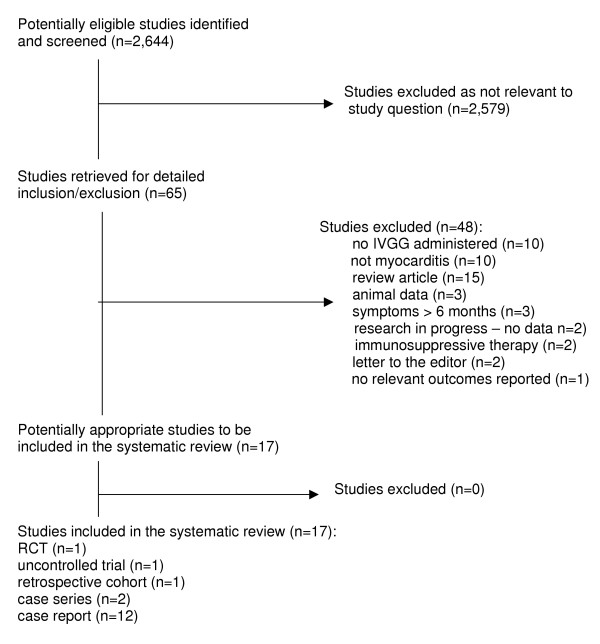
Flow diagram of studies considered for inclusion in the review

Figure 1 presents a flow diagram of studies considered for inclusion in the review. Only one RCT evaluating IVGG for acute myocarditis has been reported to date [16]. The trial was of moderate methodological quality according to the Jadad scale (3 out of maximum 5 points, where 5 indicates highest quality); allocation concealment was unclear. The study enrolled 62 adults (mean age 43.0 +/- 12.3 years; 37 men), of which only ten had cellular inflammation on endomyocardial biopsy (four fulfilled the Dallas criteria for acute myocarditis, and three for borderline myocarditis). The authors reported that the given sample size would provide 80% power to detect a difference between groups of ≥ 8% in ejection fraction (EF) change scores. Patients were randomized to receive either 2 g/kg IVGG or an equivalent volume of 0.1% albumin in a blinded fashion. The incidence of death or requirement for cardiac transplant or placement of a left ventricular assist device was low in both groups. Event-free survival was not significantly different but favored the control group (OR 0.52, 95% CI 0.12, 2.30). Follow-up at 6 and 12 months showed no significant difference in improvement in left ventricular ejection fraction for cases and controls (mean difference 0.00, 95% CI -0.07, 0.07 at six months; 0.01, 95% CI -0.06, 0.08 at 12 months). Functional capacity as assessed by peak oxygen consumption was not significantly different in the two groups at 12 months (mean difference -0.80, 95% CI -4.57, 2.97). Infusion-related side effects occurred significantly more often in the treated group (RD 0.33, 95% CI 0.17, 0.50; number needed to harm = 3, 95% CI 2, 6), but all appeared to be mild.

Sixteen additional studies of various designs met the inclusion criteria [see Additional file 1]. These included a retrospective case series [17], an uncontrolled trial [18], two case series [5,19], and twelve case reports [20-31]. In a retrospective cohort study, Drucker compared children who were treated with IVGG (n = 21) to historic controls (n = 26); 65% of the sample was less than two years of age [17]. The treatment group showed improved cardiac function and a trend (though not statistically significant) towards improved survival. McNamara conducted an uncontrolled trial involving 10 adults (mean age 35.8 +/- 15 years) [18]. One patient with refractory arrhythmias and severe heart failure died during the infusion; the other nine patients showed an impressive improvement in cardiac function following administration of IVGG, with a significant increase in left ventricular ejection fraction at 12-month follow-up (p = 0.008). In ten case reports and both case series, improvement in cardiac function was described after use of IVGG in a total of 21 cases, although improvement was considered to be minimal in one case [26]. In the other two case reports, there was no benefit of IVGG in a patient presenting with hand, foot and mouth disease and acute myocarditis from coxsackievirus A16 [27] and in a patient with fulminant myocarditis of unclear etiology [28]. Hemolytic anemia was attributed to use of IVGG in one case report [26].

We could find no ongoing trials of the use of IVGG in acute myocarditis, although the European Study of Epidemiology and Treatment of Cardiac Inflammatory Diseases (ESETCID) is evaluating hyperimmunoglobulin for patients with cytomegalovirus-positive myocarditis [32].

## Discussion

The small number of studies evaluating IVGG in the treatment of acute myocarditis reflects the immature state of this body of literature. We identified only one RCT (Level 1 evidence), which involved 62 adult patients with idiopathic cardiomyopathy (of which only 10 had histologic evidence of myocarditis) and showed no apparent benefit [9]. This is in contrast to an uncontrolled trial and numerous observational studies (Level 4 evidence), most of which suggested potential benefit. This indicates a possible bias towards publication of case reports or case series with positive results. The validity of evidence was generally poor, with the majority of included studies ranking low in terms of level of evidence. The most important threat to validity for fifteen of the seventeen included studies was the lack of controls, which could result in an overestimate of the benefit of IVGG. Spontaneous improvement in cardiac function is common with acute myocarditis and can be rapid or gradual, so it is possible that the improvement noted in these cases was part of the natural history of the disease. The retrospective cohort study demonstrated a greater improvement in cardiac function in patients given IVGG compared to historic controls [17]; this type of study is more susceptible to bias because of the inability to fully control for all potential confounders, such as other changes in patient management over the eight-year study period.

Acute myocarditis is a relatively non-specific entity, as the diagnosis is often clinical. Laboratory confirmation consists of microbiologic, histologic, and immunohistochemical methods. With regard to microbiologic confirmation, it is rare to isolate the etiologic agent from a myocardial biopsy (probably because the biopsy is usually done too late in the course of the illness), and identification of organisms by molecular techniques is in its infancy [4]. However, with the development of new effective antivirals, there should be increased efforts towards making a virologic diagnosis early in the course of myocarditis as it is possible that antivirals would improve the prognosis. Furthermore, there are case reports of hyperimmunoglobulin showing an apparent effect in cases of varicella [33], cytomegalovirus [34], and parvovirus [35] myocarditis. With regard to histologic confirmation, the Dallas criteria (lymphocyte infiltration with myocyte necrosis) [36] and more recently the World Heart Federation criteria (>14 leukocytes/mm^2 ^with necrosis or degeneration) [37] have been used for histologic diagnosis, but the sensitivity and specificity of these criteria are not known. There are no standardized values for immunohistochemical markers [38]. Our aim in this study was to analyze reports of use of IVGG for presumed viral myocarditis, but it is possible that many of the patients in the reports did not have viral myocarditis (only 10 of the 62 patients in the RTC had cellular inflammation on endomyocardial biopsy). Once our ability to accurately diagnose viral myocarditis improves, it may be possible to identify a subset of patients who will respond to IVGG. This might be patients whose disease was precipitated by a specific virus, or patients who are treated with IVGG early in the course of their illness when they have ongoing viral replication in the myocardium. Perhaps pediatric patients are more likely to respond. Children are thought to be more likely to present in the acute inflammatory stage of illness [16], and may have a worse prognosis than do adults when they present with fulminant myocarditis [39,40]

In conclusion, the value of IVGG in patients with acute myocarditis is obscured by the poor quality of evidence. We were not able to identify a subgroup of patients who appear to be more likely to respond to IVGG. A large RCT is required to evaluate the efficacy of IVGG for acute myocarditis with emphasis on the etiology of the myocarditis. Until there are RCTs demonstrating benefit, use of IVGG for acute myocarditis should not be part of routine practice. Moreover, there is a great need for further studies of the pathophysiology of acute myocarditis, which would allow for a better understanding of the etiology and the natural history of the disease. This might allow for improved diagnostic criteria, which would make it much easier to design studies of treatment options. This may also assist in identifying sub-groups of patients where IVGG or other therapies have a greater potential to confer clinical benefit.

## List of abbreviations

IVGG – intravenous gamma globulin

RCT – Randomized controlled trial

## Competing interests

The author(s) declare that they have no competing interests.

## Authors' contributions

The idea for the study originated with JLR. The protocol was written by LH with help from JLR and EC. The literature review was completed by EC, and LH and JLR reviewed the papers and derived the data. Statistical analysis was done by BV. The manuscript was written by JLR and LH with input from TPK, EC and BV.

## Pre-publication history

The pre-publication history for this paper can be accessed here:



## Supplementary Material

Additional File 1 Observational studies and nonrandomized trials examining the use of IVGG in acute myocarditis. This table summarizes all studies reporting the use of IVGG for acute myocarditis. Click here for file
