# Supplementary material for: A systematic review of intravenous gamma globulin for therapy of acute myocarditis
Source: BMC Cardiovasc Disord. 2005 Jun 2;5:12. doi: 10.1186/1471-2261-5-12 (PMC1173096; doi:10.1186/1471-2261-5-12)
Supplement: Additional File 1 — Observational studies and nonrandomized trials examining the use of IVGG in acute myocarditis. This table summarizes all studies reporting the use of IVGG for acute myocarditis. [file 1471-2261-5-12-S1.doc]

**Additional file 1**

Observational studies and nonrandomized trials examining the use of IVGG in acute myocarditis

| Author  Year  Country  Reference | Study design (Level of evidence) | N | Type of patient(s) with acute heart failure | Endomycoardial biopsy results  (number of patients) | Evidence for viral infection | IVGG regime | Outcome |
| --- | --- | --- | --- | --- | --- | --- | --- |
| Drucker 1994  United States [1] | Retrospective cohort with historical controls  (Level 4) | 46* | Children with < 3 months symptoms of which. 16/21 in IVGG group and 14/25 controls were less than 2 years of age, and 10/21 in IVGG group were males versus 14/25 controls | Definite or borderline myocarditis in 12/19 from intervention group and in 8/20 controls, no myocarditis in 7/19 from intervention group and in 8/20 controls, and no biopsy in 2/21 from intervention group and in 5/25 controls | NR | 2 g/kg as a single dose over a maximum of 24 hours with repeat 1 g/kg in 4 patients 1 week after initial infusion, with the mean time from symptom onset to admission being 2 days in IVGG group and 4.5 days in controls | Transplant-free survival at 12 months was 84% in the IVGG group versus 60% in controls (p=0.069). There was greater improvement in the LVEDD in the IVGG group than in controls at 3 to 6 months (p=0.008) and at 6 to 12 months (p=0.072) post-treatment. Mean adjusted fractional shortening was in the normal range for cases at 6 to 12 months post-IVGG (P>.10 relative to normative values), but was still below the normal range in controls (p<0.001). Left ventricular function was normal in 100% of IVGG group at 12 months, versus 37% of controls (p<0.001). No adverse effects were observed. |
| McNamara 1997  United States [2] | Uncontrolled trial  (Level 4) | 10 | Hospitalized adults with < 6 months symptoms, with NYHA class III/IV heart failure, and LVEF < 40%; of the 9 patients who completed the trial, the mean age was 35.8 (SD 15.0) years and 5 were male | Borderline myocarditis (n=1), nonspecific inflammation (n=2), no inflammation (n= 6), no biopsy (n=1) | NR | 2 g/kg over 2 to 4 days | One patient died of severe heart failure during the infusion. At 12-month follow-up in the other 9 patients, LVEF increased from a mean of 24% (SD 6%) to 41% (SD 12%) (p=0.003) and all had NYHA class I or II heart failure. There were no complications observed in the 9 patients who completed treatment and there were no rehospitalizations for congestive failure over the follow-up period (median 18 months, range 14 to 24 months). |
| Takeda 1998  Japan [3] | Case report  (Level 4) | 1 | 22 year-old female requiring percutaneous cardiopulmonary support | Borderline myocarditis | EBV detected by PCR | 2 g/kg over 2 days | Marked clinical improvement occurred over a few days and cardiac function was normal at 2-month follow-up. |
| Nigro 2000  Italy [4] | Case report (Level 4) | 1 | 7 month-old female | Diffuse lymphocytic myocarditis | Parvovirus B19 DNA was detected in serum; patient had parvovirus IgG but no IgM | 2 g/kg over 5 days | Clinical improvement and loss of viral DNA from serum occurred, but the child developed chronic persistent myocarditis which persisted at least 3.8 years. |
| Tsai 2001  Taiwan [5] | Case report  (Level 4) | 1 | 4 year-old male with complete heart block | No biopsy | Positive IgM for *Mycoplasma pneumoniae* | 2 g/kg over 48 hours about 4 days after symptom onset | Gradual clinical improvement and resolution of complete heart block to ectopic atrium heart rhythm with prolonged P-R interval occurred within 6 hours. The patientwas discharged on the 15^th^ hospital day and continued to show improved ventricular performance over 6-month follow-up period. |
| Shioji 2002  Japan [6] | Case report  (Level 4) | 1 | 31 year-old female requiring IABP | Fulminant myocarditis with massive necrosis | Negative serology for coxsackievirus and influenza | 2 g/kg over 2 days started 7 days after symptom onset | Dramatic improvement alowed removal of IABP 2 days after IVGG completed, and LVEF increased from 18% at baseline to 49% 16 days after IVGG was completed. |
| Tedeschi 2002  Italy [7] | Case report  (Level 4) | 1 | 49 year-old female | No biopsy | Negative serology for multiple organisms | 2 g/kg over 5 days starting 5 days after symptom onset | LVEF increased from 30% to 75% within 1 week of IVGG administration and the patient was in good health at 1-year follow-up. |
| Kishimoto 2003  Japan [8] | Case series  (Level 4) | 9 | Hospitalized adults with NYHA class III/IV heart failure, < 6 months symptoms and LVEF< 40%; mean age 41 years (SD 19, range 19 to 75 years); 5 males) | Myocarditis (n=4);  Nonspecific (n= 1); No biopsy (n=4) | No viruses detected in sera | 1-2 g/kg over 2 days starting 1-6 months after symptom onset (mean 2.2 months) | LVEF increased from 19 % (SD 8%) to 35% (SD 9%) a mean of 12 (SD 6) days after IVGG (P<.01) and all patients were eventually discharged and had NYHA class I or II heart failure a median of 3.5 months (range 3 to 54 months) post-IVGG. There were no subsequent rehospitalizations for congestive heart failure and no serious complications were reported. |
| Stouffer 2003 United States [9] | Case report (Level 4) | 1 | 64-year-old woman | No biopsy | Serologic evidence of acute parvovirus infection | 1 g/kg over 2 days starting about 3 weeks after symptom onset | LVEF increased from 25-30% to 55%. |
| Karaaslan 2003 Turkey [10] | Case report (Level 4) | 1 | 11-year-old boy | No biopsy | Serology negative for EBV, CMV, and parvovirus | 400 mg/kg /day for 5 days | The patient developed hemolytic anemia which was attributed to IVGG. Ejection fraction increased from 27% to 35% after IVGG, and shortening fraction increased from 12% to 17%. |
| Khan 2003 United States [11] | Case report (Level 4) | 1 | 21-day-old boy | Not done | NR | 2 g/kg 8 days after symptom onset | Shortening fraction increased from 20% 3 days prior to IVGG infusion to 33% 1 day after infusion (normal range 28-40%). |
| Wang  2004  Taiwan [12] | Case report (Level 4) | 1 | 15-month-old boy presenting with hand, foot and mouth disease | Fulminant myocarditis | Coxsackievirus A16 isolated test | 1 g/kg/day for 2 days starting 4 days after symptom onset | The patient died on the sixth day of illness because of myocardial failure. |
| Kim 2004 South Korea [13] | Case reports (Level 4) | 2 | 9-year-old and 7-year-old-girls | No biopsies | NR | 2g/kg over 10 hours | LVEF increased from 23% to 68% over 14 days in patient #1 and from 37% to 67% over 6 days in patient #2. |
| Braun 2004 Germany [14] | Case report (Level 4) | 1 | 12-year-old girl requiring biventricular assist device | No biopsy | Acute viral serology negative | 2g/kg over 24 hours | No improvement occurred until the inotrope levosimendan was added 25 days after IVGG |
| Abe 2004 Japan [15] | Case report (Level 4) | 1 | 53-year-old man requiring IABP | Fulminant myocarditis | 256-fold rise in titre for coxsackievirus B3 with no change in titre for influenza, echovirus, adenovirus, or parainfluenza | 1g/kg over 2 days | Echocardiographic improvement was noted within 2 days of IVGG infusion and the patient was weaned off IABP 5 days after IVGG infusion |
| English 2004 United States [16] | Case report (Level 4) | 1 | Child | NR | NR | 1 or 2 g/kg | Complete recovery occurred |

Legend – Table 2

IABP – intra-aortic balloon pump

LVEDD - left ventricular end-diastolic dimension

LVEF – left ventricular ejection fraction

M – myocarditis

NR – not reported

NYHA – New York Heart Association

SF – shortening fraction

* There were 25 controls and 21 cases; 5 patients received prednisone (2 in IVGG group, 3 controls)

**References**

1. Drucker NA, Colan SD, Lewis AB, Beiser AS, Wessel DL, Takahashi M, Baker AL, Perez-Atayde AR, Newburger JW: **Gamma-globulin treatment of acute myocarditis in the pediatric population.** *Circulation* 1994 ; **89**: 252-7.

2. McNamara DM, Rosenblum WD, Janosko KM, Trost MK, Villaneuva FS, Demetris AJ, Murali S, Feldman AM: **Intravenous immune globulin in the therapy of myocarditis and acute cardiomyopathy**. *Circulation* 1997; **95 :** 2476-8.

3. Takeda Y, Yasuda S, Miyazaki S, Daikoku S, Nakatani S, Nonogi H: **High-dose immunoglobulin G therapy for fulminant myocarditis.**  *Jap Circ J* 1998; **62**: 871-2.

4. Nigro G, Bastianon V, Colloridi V, Ventriglia F, Gallo P, D'Amati G, Koch WC, Adler SP: **Human parvovirus B19 infection in infancy associated with acute and chronic lymphocytic myocarditis and high cytokine levels: report of 3 cases and review*.*** *Clin Infect Dis* 2000; **31**: 65-9.

5. Tsai YG, Ou TY, Wang CC, Tsai MC, Yuh YS, Hwang B: **Intravenous gamma-globulin therapy in myocarditis complicated with complete heart block: report of one case**. *Acta Paediatr Taiwan* 2001; **42:** 311-3.

6. Shioji K, Matsuura Y, Iwase T, Kitaguchi S, Nakamura H, Yodoi J, Hashimoto T, Kawai C, Kishimoto C: **Successful immunoglobulin treatment for fulminant myocarditis and serial analysis of serum thiredoxin – a case report**. *Circulation Journal* 2002; **66**: 977-80.

7. Tedeschi A, Airaghi L, Giannini S, Ciceri L, Massari FM: **High-dose intravenous immunoglobulin in the treatment of acute myocarditis. A case report and review of the literature***. J Intern Med* 2002; **251**: 169-73.

8. Kishimoto C, Shioji K, Kinoshita M, Iwase T, Tamaki S, Fujii M, Murashige A, Maruhashi H, Takeda S, Nonogi H, Hashimoto T: **Treatment of acute inflammatory cardiomyopathy with intravenous immunoglobulin ameliorates left ventricular function associated with suppression of inflammatory cytokines and decreased oxidative stress.** *Intern J Cardiol* 2003; **91**: 173-8.

9. Stouffer GA, Sheahan RG, Lenihan DJ, Patel P, Lenihan DJ: **The current status of immune modulating therapy for myocarditis: a case of acute parvovirus myocarditis treated with intravenous immunoglobulin**. *Am J Med Sci*. **326:** 369-74.

10. Karaaslan S, Oran B, Caliskan U, Baysal T, Baspinar O, Tas A: **Hemolysis after administration of high-dose immunoglobulin in a patient with myocarditis.** *Turk J Haematol* 2003; **20:** 237-40.

11. Khan MA, Das B, Lohe A, Sharma J. **Neonatal myocarditis presenting as an apparent life threatening event.** *Clin Pediatr (Phila)* 2003; **42**: 649-52.

12. Wang CY, Li Lu F, Wu MH, Lee CY, Huang LM**: Fatal coxsackievirus A16 infection.** *Pediatr Infect Dis J* 2004; **23:** 275-6.

13. Kim HS, Sohn S, Park JY, Seo JW: **Fulminant myocarditis successfully treated with high-dose immunoglobulin.** *Int J Cardiol* 2004; **96:** 485-6.

14. Braun JP, Schneider M, Dohmen P, Dopfmer U: **Successful treatment of dilative cardiomyopathy in a 12-year-old girl using the calcium sensitizer levosimendan after weaning from mechanical biventricular assist support.** *J Cardiothorac Vasc Anest.* 2004 **18:** 772-4.

15. Abe S, Okura Y, Hoyano M, Kazama R, Watanabe S, Ozawa T, Saigawa T, Hayashi M, Yoshida T, Tachikawa H, Kashimura T, Suzuki K, Nagahashi M, Watanabe J, Shimada K, Hasegawa G, Kato K, Hanawa H, Kodama M, Aizawa Y: **Plasma concentrations of cytokines and neurohumoral factors in a case of fulminant myocarditis successfully treated with intravenous immunoglobulin and percutaneous cardiopulmonary support.** *Circ J* 2004; **68**: 1223-6.

16. English RF, Janosly JE, Ettedgui JA, Webber SA: **Outcomes for children with acute myocarditis.** *Cardiol Young* 2004; **14:** 488-93.
